# Stabilized tetraether lipids based particles guided prophyrins photodynamic therapy

**DOI:** 10.1080/10717544.2018.1482970

**Published:** 2018-07-11

**Authors:** Gihan Mahmoud, Jarmila Jedelská, Samia Mohamed Omar, Boris Strehlow, Marc Schneider, Udo Bakowsky

**Affiliations:** a Department of Pharmaceutics and Biopharmaceutics, University of Marburg, Marburg, Germany;; b Department of Pharmaceutics and Industrial Pharmacy, Faculty of Pharmacy, Helwan University, Cairo, Egypt;; c Department of Pharmaceutics, Faculty of Pharmacy, Ahram Canadian University, Giza, Egypt;; d Department of Pharmacy, Biopharmaceutics and Pharmaceutical Technology, Saarland University, Saarbrücken, Germany

**Keywords:** Tetraether lipids, liposomes, photodynamic therapy, protoporphyrin IX, chick chorioallantoic membrane

## Abstract

Photodynamic therapy (PDT) that involves ergonomically delivered light in the presence of archetypical photosensitizer such as Protoporphyrin IX (PpIX) is a time-honored missile strategy in cancer therapeutics. Yet, the premature release of PpIX is one of the most abundant dilemma encounters the therapeutic outcomes of PDT due to associated toxicity and redistribution to serum proteins. In this study, ultrastable tetraether lipids (TELs) based liposomes were developed. PpIX molecules were identified to reside physically in the monolayer; thereby the inherent π-π stacking that leads to aggregation of PpIX in aqueous milieu was dramatically improved. TEL_29.9 mol%_ and TEL_62mol%_ based liposomes revealed PpIX sustained release diffusion pattern from spherical particles as confirmed by converged fitting to Baker & Lonsdale model. Stability in presence of human serum albumins, a key element for PDT accomplishment was emphasized. The epitome candidates were selected for vascular photodynamic (*v*PDT) in *in-Ovo* chick chorioallantoic membrane. Profoundly, TEL_62mol%_ based liposomes proved to be the most effective liposomes that demonstrated localized effect within the irradiated area without eliciting quiescent vasculatures damages. Cellular photodynamic therapy (*c*PDT) revealed that various radiant exposure doses of 134, 202, 403 or 672 mJ.cm^−2^ could deliberately modulate the photo-responses of PpIX in TEL-liposomes.

## Introduction

The most widely used archetypical photosensitizer (PS), protoporphyrin IX (PpIX), exhibits extended delocalized aromatic π electron system that allows them to absorb light perfectly. Nevertheless, the π-π stacking and the hydrophobic interactions are certainly inducing aggregates in aqueous milieu, which causes limited singlet oxygen (^1^O^2^) quantum yield and low bioavailability, a shortcoming, which in turn hampers the PDT outcomes. Many attempts have been made to incorporate poorly soluble photosensitzers in conventional di-ester phospholipids containing liposomes. Nevertheless, di-ester phospholipids based liposomes often necessitate the inclusion of cholesterol to improve the rigidity of the bilayer membrane which did improve the monomerization of encapsulated photosensitizers (Vemuri & Rhodes, 1995; Lucky et al., 2015). Nevertheless, a significant protecting barrier between the incorporated photosensitizers and the surrounding milieu is yet lacking. Hence, the premature release of PS in the bloodstream before reaching the tumor site is considered as the paramount pitfall of the conventional liposomes (Derycke & de Witte, 2004). Alongside, the existence of an exchange of diester phospholipids between the liposomes and lipoproteins leads to premature disintegration of liposomes, resulting in skin photosensitivity and retinal damage, deleterious side effects that can last for 2 days to several weeks (Gilbert, 2011, Jheon et al., 2011). Conventional liposomes are also prone to opsonization by plasma proteins after which they are quickly removed from the circulation by cells of the mononuclear phagocyte system (MPS) which generally reduce the plasma half-life. Thus, they are subjected to reduced tumor cellular uptake which hampers the tumoritropic effect of PS. The dilemma of developing controlled release liposomes containing PS during blood circulation is yet to be developed wherein they could minimize its redistribution to serum proteins (Shaw & Pal, 2008). Compared to monopolar diester lipids, tetraether lipids (TELs) derived from polar lipid fraction E (PLFE) of thermoacidophilic archaeon *Sulfolobus acidocaldarius* are dominated by polyisoprenoid skeleton containing 40 carbons (C_40_) comprised two phytanyl chains. PLFE contains a mixture of caldarchaeol (glycerol dialkyl glycerol tetraether, GDGT) and calditoglycerocaldarchaeol (glycerol-dialkyl-nonitol-tetraether, GDNT). GDGT fraction has one glycerol backbone at each end of the hydrophobic core. Meanwhile, calditoglycerocaldarchaeol (glycerol-dialkyl-nonitol-tetraether, GDNT) is attached to a glycerol backbone at one end of the hydrophobic core and a calditol group at the other end (Chong et al., 2012; Wang et al., 2012). The polyisoprenoid skeleton is linked to two polar head groups through ether bonds, by which they are arranged as a monolayer in the cytoplasmic membrane. In addition, the unique molecular structure of TELs is attributed to the presence of cyclopentane rings, methyl side groups and sugar moieties that create extensive hydrogen bond network. The presence of ether bonds gives rise to molecular stability (Engelhardt et al., 2017), in addition to the lacking of double bonds which make TELs an epitome candidate for PDT. One of the elementary factors for tumor progression, for instance, growth, invasion and metastasis, is angiogenesis. The damage to the microvasculature and suppression of angiogenesis is supposed to provide potent modality for solid tumor ablation which in turn, induce tissue necrosis by anoxia (Dudek et al., 2003, Johansson & Andersson-Engels, 2010; Shi et al., 2017). The developing chicken embryo is surrounded by a transparent chorioallantoic membrane (CAM), which becomes highly vascularized as the embryo develops. The structural changes of individual blood vessels in the transparent membrane could be previously examined in real time (Makanya et al., 2016). The study of the photothrombic effects of *v*PDT were also evaluated after topical application (Toledano et al., 1998; Hammer-Wilson et al., 1999), direct injection into the yolk sac (Gottfried et al., 1995) or intravenous injection of PS into CAM blood vessels (Vargas et al., 2004; Pegaz et al., 2006). Shortly after PS administration, the so-called short drug-light interval (DLI), PS accumulates passively in the vascular compartment after which vascular-targeted PDT is performed. Whereas, long DLIs resulted in the accumulation of PS in the extravascular compartment of the tumor, due to leakage from the vasculature and interstitial diffusion (Dolmans et al., 2003; Li & Luo, 2009, Johansson & Andersson-Engels, 2010). Unlike PDT studies in animal models, the irradiation of transparent CAM with an appropriate wavelength is feasible and the light tissue-penetration is not a key determined step. The potential uses of TELs were previously comprehensively evaluated as a platform for photodynamic therapy at our laboratory (Mahmoud et al., 2015, 2017). In this study, TELs were assembled to form highly stabilized liposomes prevailing sustained release pattern. The combination of short interval PDT (vascular targeting) *in-Ovo* chick CAM model and long interval PDT (cellular targeting) in SKOV-3 and L929 cell lines was studied.

## Materials and methods

### Materials

The polar lipid fraction was obtained from *Sulfolobus acidocaldarius* (Surface and Interface Technologies (SIT) Rosenhof GmbH, Heiligenstadt, Germany). Protoprophyrin IX (PpIX), Human Serum Albumin (HSA, average MW = 67,000) and octyl β-D-glucopyranoside (OGP) were purchased from Sigma-Aldrich Chemie GmbH (Steinheim, Germany). 1,2-dipalmitoyl-sn-glycero-3-phosphocholine (DPPC) was obtained from Lipoid GmbH (Ludwigshafen, Germany) and was used without any further purification.

### Preparation of PpIX containing TEL-liposomes

Various mole fraction combinations of di-ester phospholipid 27.2 × 10^−4^ to 11 × 10^−3 ^M (DPPC) and tetraether lipids 11.24 × 10^−4^ to 4.5 × 10^−3 ^M (TELs) were used to prepare TEL_9mol%,_ TEL_29mol% and_ TEL_62mol%_ liposomes. Total lipid (TL) to PpIX mass ratios of 10 and 100 were set for each formula. The liposomes were prepared using thin-film hydration technique as previously reported (Mahmoud et al., 2015). Briefly, the appropriate amounts of TELs, DPPC and PpIX were diluted in a mixture of chloroform: methanol (2:1v/v). The organic solvent was subsequently evaporated under an escalating vacuum at 150 rpm as follows; 800 mbar for 3 seconds, 300 mbar for 3 min and 2 mbar for 60 min using the rotary evaporator Heidolph Laborota 4000 efficient (Heidolph Instruments, Schwabach, Germany). The temperature was held slightly above the phase transition temperature of the dominant lipid in mixture at 50 °C until a thin film was deposited. The crude multi-lamellar vesicles (MLV) were formed after addition of HEPES-buffered saline to the formed thin film and subjected to five freeze-thawing cycles. One cycle was to freeze the liposomes in liquid nitrogen for 5 min followed by thawing in water bath at 50 °C for further 5 min using bath sonication at 100% amplitude. For constraining particle size distribution, the obtained liposomes were subjected to extrusion process using polycarbonate filter of pore size 200 nm at 50 °C.

### Characterization of TEL-liposomes

#### Photophysical properties of PpIX in TEL-liposomes

Fluorescence spectra of the previously prepared PpIX in TEL-liposomes were studied. Liposomes were diluted at the time of measurement in HEPES-saline buffer at pH = 7.4. The emission spectrum of PpIX was measured using a Perkin Elmer LS50-B fluorescence spectrometer at λ_em_ 640 nm (λ_ex_ 408 nm). Excitation and emission bandwidths were set to 10 nm at scan rate of 100 nm/min. Five accumulated scans of the steady-state emission spectra of free PpIX in ethanol, DPPC (TEL_0mol%_), TEL_9mol%,_ TEL_29mol%_ or TEL_62mol%_ at TL: PpIX ratios of 10 and 100 were reported.

#### Physicochemical properties of TEL-liposomes

The hydrodynamic diameters of TEL-liposomes were measured using Dynamic Light Scattering (DLS) (Zetasizer Nano ZS (Malvern Instruments, Herrenberg, Germany) equipped with a 10 mW HeNe laser at wavelength of 633 nm. Scattered light was detected at an angle of 173°. The zeta potential values were determined using Laser Doppler Velocimetry (LDV), where the scattered light is collected at an angle of 17°. The obtained results were presented as an average value ± standard deviation of three independent preparations with three replicate measurements of each preparation for at least 10 runs.

#### Cryo scanning electron micrographs of TEL-liposomes

TEL-liposomes were investigated under cryogenic conditions using a 400 kV JEM-4000SFX (JEOL, Japan) Cryo-scanning electron microscope operating at 4.2 K. Briefly, the prepared TEL-liposomes were cooled down in liquid nitrogen to -95 °C. The frozen liposomes were then subjected to ice sublimation step for 15 minutes. The liposomes were further cooled down at -140 for 10 minutes before they were sputter coated.

### Redistribution of PpIX from TEL-liposomes to PpIX-free DPPC liposomes or human serum albumin (HSA) acceptors

Referring to complete PpIX quenching in TEL-liposomes prepared at TL: PpIX mass ratio of 10, they were selected for PpIX redistribution study (Reshetov et al., 2011). The transfer of PpIX in TEL-liposomes (TEL_PpIX-Donor_) to PpIX-free DPPC (DPPC_PpIX-Acceptor_) or HSA acceptors (HSA_PpIX-Acceptor_) was undertaken. Ten millimolar of DPPC_PpIX-Acceptor_ unilamellar acceptor liposomes were prepared as described earlier for liposomes preparation excluding the addition of TELs and PpIX. TEL_PpIX-Donor_ was diluted at an identical final PpIX concentration at 22.2 × 10^−6^ M. Aliquots of TEL_PpIX-Donor_ were incubated at 37 °C with DPPC_PpIX-Acceptor_ equivalent to 20-folds DPPC_PpIX-Acceptor_: TEL_PpIX-Donor_ molar ratio of lipids or with HSA_PpIX-Acceptor_ at 200 µM to accomplish PpIX distribution. The change in fluorescence was subsequently monitored at λ_em_ 640 nm (λ_ex_ 408 nm) during incubation. Data of release was calculated as PpIX fluorescence recovery [%], from the normalized fluorescence due to the dilution of PpIX among TEL_PpIX-Donor_ and the acceptors according to equation (1):

(1) $$\text{Fluorescence recovered}\left[\text{\%}\right]=\left[\frac{\Delta Ft\;–\;Fo}{FOGP}\right]\times 100\text{\%}$$

where (F_t_) is PpIX fluorescence intensity measured at certain incubation time points. The fluorescence at time t (F_t_) was corrected for the initial value (F_o_) obtained prior to addition of DPPC_PpIX-Acceptor_ or HSA_PpIX-Acceptor_. F_OGP_ denotes the final de-quenching of PpIX after complete dilution using 20 mM OGP at the end of the time course. The normalized fluorescence values obtained during incubation correspond to the sum of normalized fluorescence values of (Δ_PpIX-Donor_) and acceptor liposomes (Δ_PpIX-Acceptor_). Hence, independent measurements were derived based on the normalized fluorescence of TEL-liposomes prepared at different TL: PpIX ratios, from which the corresponding TL: PpIX ratios at each time was calculated. The obtained results were presented as an average value of at least three replicates ± Standard Deviation. Difference factor ($f_{1}$), as a pairwise procedure, was calculated to study the dissimilarity between the release profiles of PpIX from TEL_29.9 mol%_ and TEL_62mol%_TEL-liposomes, compared to that from TEL_9mol%_ liposomes as previously reported (Moore, 1996; Gohel et al., 2009) using equation (2).

(2) $$f_{1}=\left[\frac{\left[\sum_{t=1}^{n}\left|R_{t}-T_{t}\right|\right]}{\sum_{t=1}^{n}R_{t}}\right]\times 100$$

where $f_{1}$ calculates the percent difference between the two curves at each time point and measures the relative error between the two curves, n is the number of time points, $R_{t}$ is PpIX released value of the reference at time *t*, and $T_{t}$ is the released value of the test at time t. Value for $f_{1}$ more than 15.0 shows dissimilarity of the two profiles.

### Mathematical kinetic analysis of PpIX transfer data

Release kinetics and mechanism of PpIX from various TEL_PpIX-Donor_ were studied. The transfer data obtained was fitted to various mathematical models (zero, first order), Higuchi diffusion and Baker–Lonsdale models. The data were also analyzed using Korsmeyer–Peppas model. Regression analysis was performed and the best fitting data were calculated on the basis of correlation coefficient, *r*^2^.

### Cell culture experiments

#### Cell conditions

The mouse fibroblast, L929 and the human ovarian carcinoma, SKOV-3 cells were obtained from ATCC (American Type Cell Culture, Manassas, USA). L929 were cultivated in a high glucose Dulbecco’s Modified Eagle’s Medium (DMEM) (Biochrom GmbH, Berlin, Germany) at 37 °C and 8% CO_2_ under humid conditions. SKOV-3 were cultivated in Iscove's Modified Dulbecco's Medium (IMDM) (Biochrom GmbH, Berlin, Germany) at 37 °C and 7% CO_2_ under humid conditions. Both the media were supplemented with 10% fetal calf serum (PAA, Cölbe, Germany).

#### Cellular photodynamic therapy (cPDT)

At the time of passage, cells were seeded into 96-well plates (NUNC, Thermo Scientific^TM^, Germany) at cell density of 25,000 cells. cm^−2^. After stabilization for 24 h, the cells were treated with TEL-liposomes at PpIX = 780 nM–100 μM; TELs = 0, 9 or 62 mole% at TL: PpIX ratio of 10. After long PpIX-light interval (t_PpIX_ = 3 h), PpIX was illuminated inside the cells at the Q-band 630 nm.

#### Light delivery

A prototype light emitting diode (Lumundus GmbH Eisenach, Germany) adopted for 96 well plates, providing irradiance (I) of 22.4 W.m^−2^ at 625 nm was used. The radiant exposure dose (D) in J.m^−2^ equal to irradiance (I) in W.m^−2^ times the time of exposure (t) in seconds (Kochevar & Anderson, 1983). Different exposure times of 60, 90, 180 and 300 s were applied and hence the cells were receiving radiant exposure doses of 134, 202, 403 or 672 mJ.cm^−2^, respectively thence, cells were further incubated for 24 h.

#### MTT cell viability assay

Cytotoxicity after *c*PDT was determined by measurement of cell viability based on the cellular redox potential as previously reported (Mosmann, 1983). Briefly, the medium was aspirated and 3-(4,5-Dimethylthiazol-2-yl)-2,5-Diphenyltetrazolium Bromide (MTT) reagent was subsequently added. Cells were further incubated for 4 h in the dark. Actively respiring cells convert the water-soluble MTT to an insoluble purple formazan. The formazan was then solubilized in DMSO and its concentration was determined at 570 nm using a plate reader (FLUOstar, BMG, Germany). Five wells per dilution were averaged, and all experiments were run in triplicate. Wells containing culture medium, but no cells were used as blank value and untreated cells were used as control representing 100% viability. The viability of the tested cells was calculated using equation (3).

(3) $$\text{Viability \%}=({\text{Ab}}_{\text{Sample }}-{\text{ Ab}}_{\text{Blamk}})/({\text{Ab}}_{\text{Control }}-{\text{ Ab}}_{\text{Blank}})\text{ × }100\text{\%}$$

where Ab_Sample_ and Ab_Control_ denote the optical density at 570 nm of the produced formazan in actively respired treated and untreated cells, respectively. Half-maximal inhibitory concentrations (IC_50_) for PpIX (PpIX_IC50_) were calculated from the fitted dose-response curves using OriginPro 8 software (OriginLab Corporation, Northampton, Massachusetts), for direct comparison of PpIX photo responsiveness among different TEL-liposomes and under comparable radiant exposure doses. Results were expressed as mean ± standard deviation (SD) of quintuplicates (*n* = 5).

#### Measurement of cellular singlet oxygen species (cROS) generation

To measure cROS formation after cPDT treatment, the cell permeant reagent 2′,7′-dichlorofluorescin diacetate (DCFDA, Sigma), for ROS detection was used according to Abcam (Cambridge, UK) protocol. The deacetylated form of DCFDA is later oxidized by ROS into 2′, 7′-dichlorofluorescein (DCF) that emits a detectable green fluorescence. Briefly, SKOV-3 cells were incubated at a final DCFDA concentration of 25 μM in phenol red-free medium for 45 min at 37 *°*C. After single washing step with Ca^+2^ and Mg^+2^ containing PBS buffer, aliquots of TEL_9mol%,_ or TEL_62mol%_ liposomes equivalent to PpIX at final concentration of 25 µM was added and incubated (t_PpIX_ = 3 h). After *c*PDT treatment at radiant exposure doses of 134, 202, 403 or 672 mJ.cm*^−^*^2^, the fluorescence was subsequently measured in the cellular lysate at λ_em_ 520 nm (λ_ex_ 485 nm) using a plate reader (FLUOstar, BMG, Germany). Five wells per dilution were averaged, and all experiments were run in triplicate.

### Confocal laser scanning microscope

The cellular uptake of PpIX in TEL-liposomes in L929 cells was evaluated using CLSM. At the time of passage, L929 cells were grown at cell density of 0.1 × 10^6^ cells. cm^−2^ on 18 × 18 mm sterile cover glasses, inserted in a 12-well plate at 37 °C for 18 h. Free PpIX or PpIX in TEL_9mol%_ or TEL_62mol%_ liposomes were diluted in a complete medium at final concentration of 25 μM PpIX. The cells were incubated for 1 or 3 h at 37 °C. After the medium was removed, cells were washed with Ca^+2^ and Mg^+2^ containing PBS buffer and subsequently were fixed for 10 min with 4% paraformaldehyde solution at room temperature. The cell nuclei were stained by addition of 4',6-diamidino-2-phenylindole (DAPI) at 3.63 mM for 2 min. After washing, the cover glasses were transferred onto glass slides for imaging with a confocal laser-scanning microscope (Zeiss, LSM 510, Germany). DAPI fluorescence was excited by a UV laser at 364 nm and detected after passing through 385–470 nm band pass filter. PpIX was excited by argon laser at 514 nm and the red fluorescence emission of the samples was detected after passing 585 nm long-pass filters. Laser intensities and detector gains were kept similar.

### Vascular-targeted photodynamic therapy (vPDT) after short PpIX-light interval

Fertilized specific pathogen-free (SPF) eggs were received from VALO BioMedia GmbH (Osterholz-Scharmbeck, Germany). Intact chick CAM angiogenesis model was prepared as reported elsewhere (Ozcetin et al., 2011). On day 11 of embryo development (EDD 11), a small Teflon ring (diameter 5 mm, wall thickness 0.5 mm, height 1 mm) was placed on the surface to define the treated area. Prior to the injection, an image of CAM surface was recorded. Subsequently, equivalent volume to 20 µM free PpIX or PpIX in TEL-liposomes was injected intravenously *in-Ovo* under a Stereo Microscope (Zeiss Stemi 2000-C, Carl Zeiss, Jena, Germany) and the circulation was observed at magnification of 12.5 X. Following the homogenous distribution of the photosensitizer in the blood circulation, *v*PDT was performed using Laser Diode Lamp at 634 nm, 4 mW using Weber Needle® Endolaser (Weber Medical GmbH, Lauenförde, Germany). The irradiation was performed continuously for 2 minutes to an area of 3.14 mm^2^. The laser power was adjusted to 100% (5.2 mW) providing radiant energy of 19.9 J.cm^−2^. After laser irradiation, eggs were returned to the incubator in a static position and the response of vascular occlusion was monitored at different time intervals of 5, 30, 60 and 120 min postirradiation (t_post_) using a 5.0 Mega Pixel digital signal camera (Moticam 5) (Motic Asia, Hong Kong). Microscopic examinations were carried out at each time point after *v*PDT and the survival rates were recorded. For each liposomal preparation, the experiment was performed at least three times. Acquisition of images was made pre- and post- *v*PDT and were compared and graded according to the criteria described elsewhere (Pegaz et al., 2006; Lim et al., 2010). Briefly, the response was graded depending on the degree of the vascular occlusion and/or destruction of CAM after *v*PDT, which representing the level of treatment. Yet, the onset of vascular response was precisely taken into consideration.

### Statistical analysis

Two-way analysis of variance (ANOVA) for the comparisons of mean values was applied using the Statistical Package for Social Sciences (SPSS) version 20 (IBM SPSS, Inc, Chicago IL). For multiple comparisons, *post hoc* tests with Dunnett’s (multiple comparisons against a control group) or Tukey’s (all pairwise comparisons) were applied. Significance levels for rejection of the null hypothesis were considered at *p* ≤ .05.

## Results and discussion

### Characterization of PpIX-loaded TEL-liposomes

#### Photophysical properties

**Figure 1.**
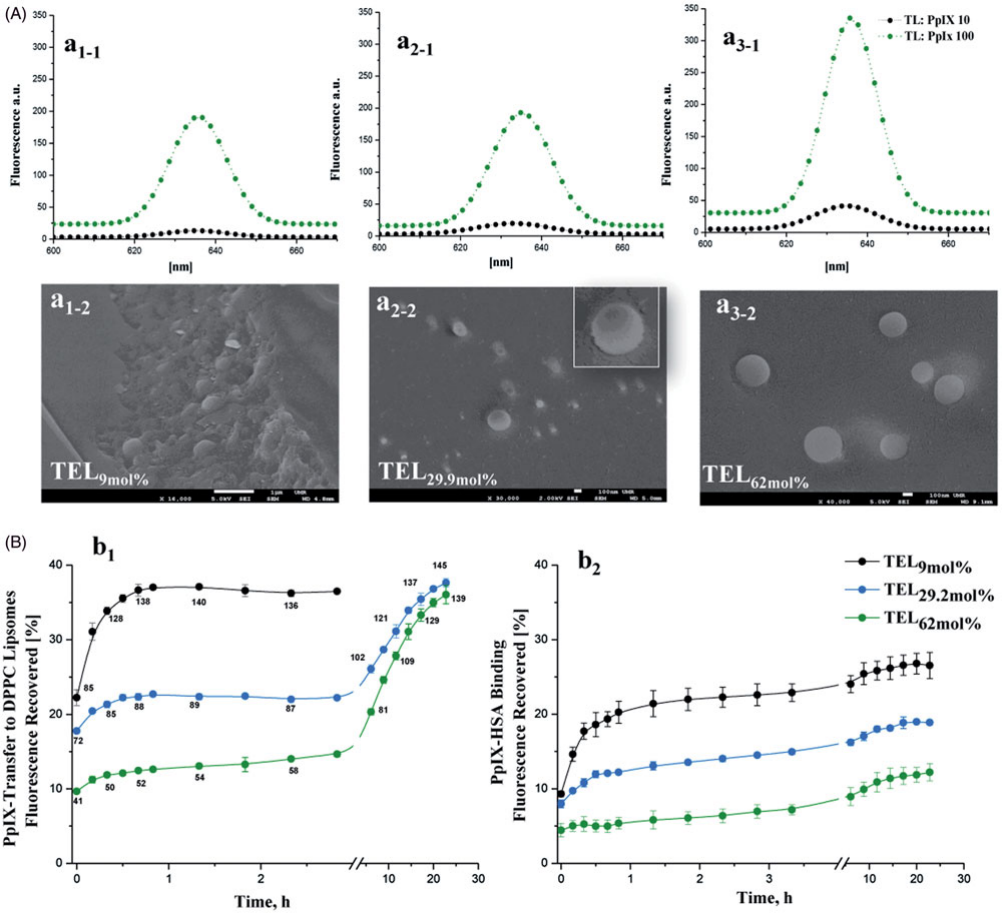
Fluorescence spectra of PpIX in various TEL-liposomes and their freeze-fractured micrographs (A). The fluorescence of PpIX in TEL_9mol%_ (a_1_), TEL_29.9 mol%_ (a_2_) and TEL_62mol%_ (a_3_) based liposomes for TL: PpIX 10 (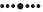 and 100 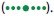 Freeze-fracture micrographs of PpIX manifested well-segregated vesicular structures in TEL_9mol%_, (a_1-2_), TEL_29.9 mol%_ (a_2-2_) and TEL_62mol%_(a_3-2_) based liposomes. Part B illustrates the *in-vitro* time-dependent fluorescence recovery of PpIX from TEL_PpIX-Donor_ based TEL_9mol%,_ (black solid circles), TEL_29.2 mol%_ (blue solid circles) or TEL_62mol%_ (green solid circles) liposomes after incubation with DPPC_PpIX-Acceptor_ liposomes (b_1_) or HSA_PpIX-Acceptor_ (b_2_). Values at the curves stand for the calculated TL: PpIX ratios during PpIX transfer. Data were shown as mean ± standard deviation of three independent experiments.

The π-π stacking and the hydrophobic interactions of PpIX induce self-association and formation of aggregated species in aqueous milieu, that exhibit no or only negligible photodynamic activity (Scolaro et al., 2002). To overcome the aforementioned limitation, many liposomal formulations have been previously utilized to enhance monomerization of photosensitizers and to restore the optimal PDT outcome (Reshetov et al., 2011). For the first attempts, tetraether lipids (TELs) were utilized to incorporate PpIX and to improve the photodynamic outcomes. TEL-liposomes were prepared at various concentrations of TELs at total lipid (TL) to PpIX mass ratios of 10 and 100 at which PpIX is completely quenched or monomerized, respectively. Based on vesicles (Lelkes et al., 1983), two models were postulated. TEL_9mol%_ liposomes, a tetraether lipids-poor membrane, impart minimal membrane stability compared to TEL_29.9 mol%_ and TEL_62.9 mol%_ liposomes, a tetraether lipids-rich membrane. As a first gauge of relative photo-physical properties of PpIX, the influence of various TEL-liposomes on aggregation/monomerization status was proved (Figure 1(A)). The steady-state emission spectra of free PpIX in ethanol and in TEL-liposomes composed of TEL_9mol%_ (Figure 1(a_1-1_)), TEL_29.9 mol%_ (Figure 1(a_2-1_)) or TEL_62mol%_ (Figure 1(a_3-1_)) at TL: PpIX ratios of 10 and 100 are depicted. Principally, the incorporation of PpIX in TEL-liposomes induced substantial increase in its emission value, which can be merely attributed to changes in the aggregate/monomer equilibrium. The bathochromic shift of the emission maxima of PpIX from 633 nm to 636 nm in ethanol (data not shown) and TEL-liposomes, respectively, was simultaneously observed regardless to TL: PpIX ratios. As the fluorescence properties of PpIX in liposomes primarily depend on the local dye concentration relative to TL in liposomes, an increase in PpIX content was accompanied by a decrease in fluorescence, with almost complete quenching at TL: PpIX ratio of 10. This, in turn, indicates a strong fixation of the adjacent molecules of PpIX in the lipid mono/bilayer which most probably dissipates the energy migration among PpIX molecules, a phenomenon called self-quenching effect (Kachatkou et al., 2009; Reshetov et al., 2011; Huynh & Zheng, 2014). The gradual appearance of the emission band at 633–36 nm (peak of the monomer) with increasing TL: PpIX ratio from 10 to 100 and, in a similar study, from 50 to 500 confirmed PpIX monomerization as TELs incorporated in liposomes (Mahmoud et al., 2017).

**Figure 2.**
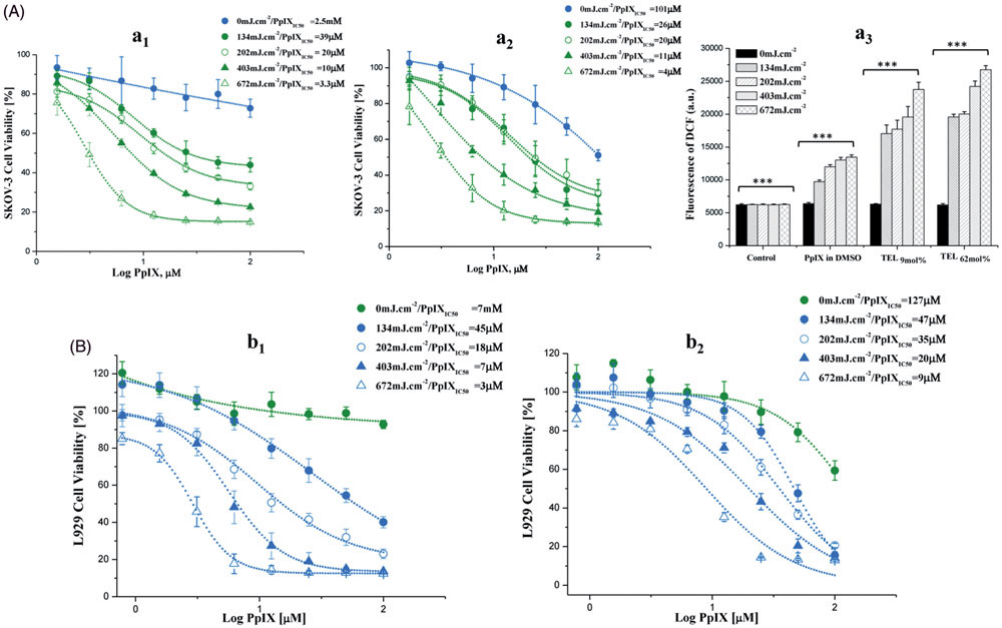
Photo cytotoxicity and dose-dependent effect of PpIX in SKOV-3 (A) and L929 (B) cells. After incubation with TEL_9mol%_ liposomes (a_1_ and b_1_) or TEL_62mol%_ (a_2_ and b_2_) liposomes at TL: PpIX 10 (t_PpIX_ = 3h), cells were light irradiated at a series of radiant exposure doses of 134, 202, 403 and 672mJ.cm^−2^ (irradiance = 22 W.m^−2^). Cell survival is represented as a percentage of control-cell growth in cultures containing no PpIX. Cell proliferation was quantified colorimetrically using MTT assay. Half-maximal inhibitory concentrations (IC_50_) for PpIX were calculated from the fitted dose–response curves. In graph a_3_, elevated generation of singlet oxygen in SKOV-3 after various *c*PDT treatments with PpIX at the same radiant exposure doses is presented. Each point represents the mean of quintuplicates (*n* = 5) ±SD of three separate experiments; ***p < .001.

**Figure 3.**
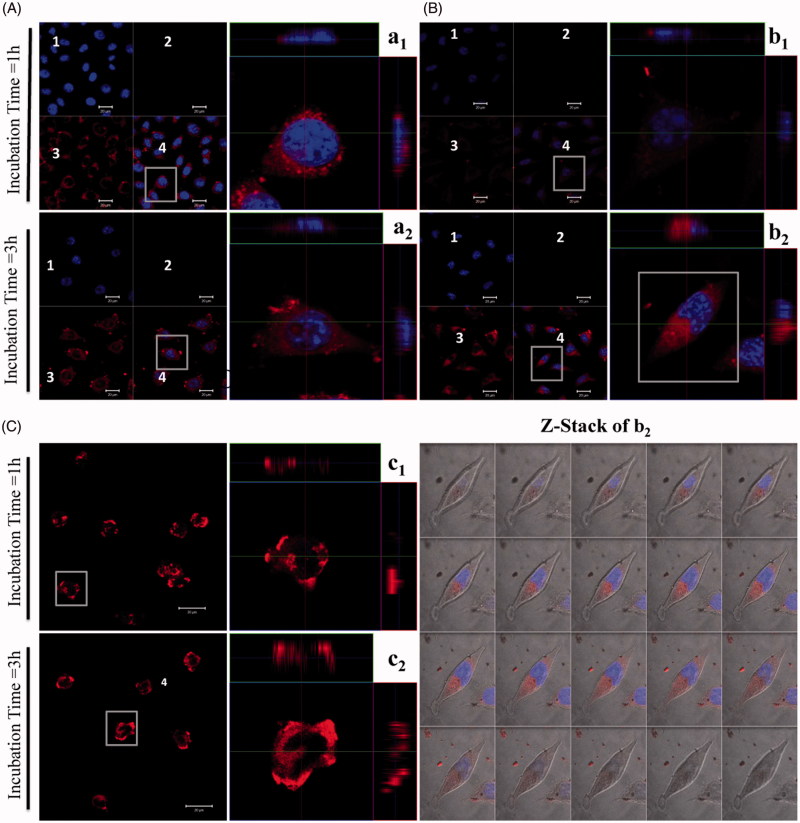
Confocal laser scanning acquisitions of L929 cells treated with various TEL-liposomes at PpIX =25 µM. Image acquisitions of TEL_9mol %_ liposomes (a_1(t=1h)_, a_2(t=3h)_) and TEL_62mol %_ liposomes (b_1(t=1h)_, b_2(t=3h)_) and free PpIX (c_1(t=1h)_, c_2(t=3h)_) are presented. (1) DAPI localized nuclei images, (2) empty channel, (3) PpIX fluorescence images and (4) merged images (magnified, right panel). Z-Stack mode acquisition of b_2_ shows the cellular co-localization of PpIX in the cytoplasm after incubation with TEL_62mol%_ liposomes (t_PpIX_=3h). The total stack size was 9.5 μm with scale of 0.5 μm each.

#### Physicochemical properties of PpIX loaded TEL-liposomes

The particle size analysis of TEL-liposomes showed no wide differences in terms of the mole fraction of TELs and TL: PpIX ratios of 10 and 100 as presented in Table 1. As TELs and PpIX carry net negative charges, their incorporation in liposomes resulted in negative zeta potential values. Liposomes composed of DPPC contain neither TELs nor PpIX demonstrated zeta potential value of about +3.03 mV. The addition of TELs to PpIX-free liposomes revealed higher zeta potential values (−20.5 mV, −34.7 mV and −40.3 mV) for TEL_9mol%_, TEL_29.9 mole%_ and TEL_62mole%_, respectively. The zeta potential values showed further increase after addition of PpIX, resulted in the highest zeta potential values for TL: PpIX ratio of 10 in all liposomes.

**Table 1.** Particle size and zeta potential measurements of TEL-liposomes.

TEL _mol%_ in liposomes (TL: PpIX ratios)		Particle size ± SD nm (PDI)		Zeta-potential ± SD mV
TEL_0mol%_				
PpIX-free liposomes		172.3 ± 1.3 (0.1)		+3.0 ± 0.1
(100)		134.2 ± 0.6 (0.1)		−22.9 ± 3.0
(10)		186.3 ± 3.6 (0.3)		−42.0 ± 3.3
TEL_9mol%_				
PpIX-free liposomes		169.4 ± 2.9 (0.2)		−20.5 ± 0.6
(100)		139.3 ± 1.4 (0.1)		−34.1 ± 1.5
(10)		173.0 ± 2.6 (0.3)		−38.3 ± 4.1
TEL_29.9mol%_				
PpIX-free liposomes		207.1 ± 18.0 (0.3)		−34.7 ± 3.52
(100)		142.6 ± 0.4 (0.1)		−36.1 ± 1.5
(10)		256 ± 6.1 (0.3)		−41.9 ± 2.17
TEL_62mol%_				
PpIX-free liposomes		194.0 ± 3.0 (0.4)		−40.3 ± 0.5
(100)		163.5 ± 2.0 (0.2)		−39.8 ± 3.9
(10)		176.5 ± 2.4 (0.2)		−44.1 ± 0.2

#### Morphological hierarchies of TEL-liposomes

The morphological hierarchies of TEL-liposomes revealed fracture plane in TEL_9mol%_ liposomes (Figure 1(a_1-2_)), which was lacked in TEL_29.9 mol%_ and TEL_62mol%_ as depicted in Figure 1, a_2-2_ and a_3-2_, respectively. Addition of DPPC promotes curvature of the bilayer in order to form closed vesicles (Chong, 2008). The liposomal membrane structure made from di-ester phospholipid and bipolar ether based phospholipids demonstrated preferential orientation of the monopolar diester phospholipid molecules toward the outer face of the lipid membrane in bipolar ether-rich vesicles (Lelkes et al., 1983).

### PpIX redistribution from TEL-liposomes and stability in human serum albumin

Due to the self-quenching property of PpIX molecules at TL: PpIX 10, the energy that normally released to fluorescence is dissipated elsewhere (Reshetov et al., 2011, Huynh & Zheng, 2014). The transfer of lipophilic molecules, including PpIX, through membranes is occurring as a result of collision among liposomal membranes according to their concentration gradient from the donor to the acceptor compartments. Investigations in this context were therefore focused on monitoring the fluorescence restoration of PpIX from TEL_PpIX-Donor_ after its distribution to DPPC_PpIX-Acceptor_ or HSA_PpIX-Acceptor_ (Reshetov et al., 2011). The release pattern varied according to the mole fraction of TELs incorporated in liposomes (Figure 1(B)). For instance, a high PpIX fluorescence recovery in case of TEL_9mol%_ liposomes was detected at the onset of incubation with DPPC acceptor (Figure 1(b_1_)). The burst transfer rate of PpIX within the first 2 h of incubation showed an increase equivalent to ≈35% of its corresponding donor liposomes. Nevertheless, TEL_29.2 mol%_ and TEL_62mol%_ demonstrated slower increase in PpIX fluorescence recovery during the first 2 h of incubation as only 22% and 13%, respectively, after which the rate of release was much faster led to a biphasic distribution pattern (Figure 1(b_1_)). Since the values of the recovered fluorescence are directly related to PpIX concentration in liposomes, we calculated TL: PpIX ratios at each incubation time point, using the method described in the experimental section. The calculated *f*1 values for TEL_29.2 mol%_ and TEL_62mol%_ liposomes revealed high value difference that constitutes 26 and 45 for TEL_29.2 mol%_ and TEL_62mol%_ liposomes, respectively compared to TEL_9mol%_ liposomes taken as a reference signifying dissimilarity in the release profiles. PpIX binding to HSA was subsequently monitored at the selected time intervals (Figure 1(b_2_)). The distribution of PpIX from TEL_PpIX-Donor_ to HSA_PpIX-Acceptor_ showed similar dependency on the mole fraction of TELs in liposomes. An increase in PpIX fluorescence to 23% was observed after 3 h of incubation with TEL_9mole%_ liposomes; whereas 15% and 7% in case of TEL_29.9 mol%_ and TEL_62mol%_ liposomes, respectively. The calculated *f*1 values of 33 and 64 for TEL_29.2 mol%_ and TEL_62mol%_ liposomes, respectively, taken TEL_9mol%_ liposomes as a reference. In serum, the ether bonds and the spanned TELs provide better stability compared to other di-ester phospholipids. This may be evaluated as evidence that ether-based liposomes have a resistance against high-density lipoproteins. The stability of TELs based liposomes in conditions mimicking the biological fluids under highly acidic conditions (pH 2) and in the presence of fetal calf serum, has been previously demonstrated (Benvegnu et al., 2005; Benvegnu et al., 2008). The incorporated PpIX is partially shielded from high incidence of collision with DPPC_PpIX-Acceptor_ or HSA_PpIX-Acceptor_ as a function of TELs. The low solute permeation in and out TELs monolayer membrane has been raised to the extensive hydrogen bonds around the polar head groups of TELs due to sugar moieties. Hence, there is a reasonable electrical dipole potential, which minimizes the solute permeation among the voids in the monolayer (Chong, 2008). Profoundly, the covalently linked biphytanyl chains to polar head groups on each side are exhibiting limited gauche-trans isomerization by which a low rate motion for solute permeation is possible (Chong, 2008). The release data shows best fitting to first order pattern based on correlation coefficient, *r^2^*values ∼1 as shown in Table 2. The transfer mechanism of PpIX from TEL_9mole%_ liposomes revealed the highest correlation coefficient according to Higuchi diffusion model. The transfer mechanism of PpIX from TEL_29.2 mole%_ or TEL_62 mol%_ was obviously fitted to Baker Lonsdale model (*r*^2^ ≈ 1), which describes the diffusion of controlled released drugs from spherical particles (Chien, 1988). The transfer data of PpIX from TEL_62mol%_ liposomes followed Fickian diffusion exponent (*n*) of 0.40; which is the limiting value for release from spheres (Ritger & Peppas, 1987; Wang et al., 2015).

**Table 2.** *In vitro* PpIX release kinetics and mechanisms from TEL-liposomes.

		Kinetic of PpIX Release						Mechanism of PpIX Release								
Kinetic Modesl (Dash et al., 2010)		Zero Order		** **	First Oder			Diffusion (Higuchi Model)			Baker & Lonsdale			Korsmeyer–Peppas		
*In-Vitro* PpIX release kinetics and mechanisms from TEL-liposomes (donor) to PpIX-free DPPC liposomes (acceptor)																
Kinetic Equation		$M_{t}/M_{\infty }=K_{0}t$			$\ln\;(1-M_{t}/M_{\infty })=-K_{1}t\;$			$M_{t}/M_{\infty }=K_{H}t^{1/2}$			$3/2\;\left[1-\left(1-\right.\right.\left.M_{t}/M_{\infty }\right)\left.2/3\right]$$-M_{t}/M_{\infty }=\;K_{BL}t$			$M_{t}/M_{\infty }=K_{KP}t^{n}$		
Kinetic Parameters		*r^2^*	*K_0_*		*r^2^*	*K_1_*		*r^2^*	*K_H_*		*r^2^*	*K_B&L_*		*r^2^*	*n*	*Kkp*
TEL9 mol%		0.88393	15.82463		**0.89597**	0.22856		**0.98241**	16.56128		**0.91763**	0.02055		0.99040	**0.11200**	38.17684
TEL29.9 mol%		0.99110	0.81309		**0.99347**	0.01158		**0.99102**	4.78710		**0.99221**	0.00101		0.94090	**0.21090**	18.90166
TEL62 mol%		0.98729	1.18327		**0.99199**	0.01577		**0.99714**	7.03667		**0.99624**	0.00118		0.98330	**0.40390**	10.26124
*In-Vitro* PpIX release kinetics and mechanisms from TEL-liposomes (donor) to HSA (acceptor)																
TEL9 mol%		0.95042	0.25853		**0.95249**	0.00342		**0.98518**	1.57791		**0.95789**	0.00025		0.99280	**0.08520**	20.80176
TEL29.9 mol%		0.92609	0.34173		**0.93003**	0.00405		**0.98036**	1.91829		**0.94822**	0.00020		0.99420	**0.13450**	12.67651
TEL62 mol%		0.95862	0.35997		**0.96077**	0.00394		**0.99045**	1.85219		**0.97866**	0.00011		0.93020	**0.21780**	5.96624

M_t_/M_0_: The fraction of drug released at time t; K_0_: Zero order release rate constant; t: The release time; KO_1_: First order release rate constant; r^2^: Correlation Coefficient Squared; K_H_: Higuchi release rate constant; n: The parameter that depends on the release mechanism; K_B&L_: Baker & Lonsdale release rate constant; KKP: Korsmeyer–Peppas release rate constant.

### Photo responsiveness of PpIX in TEL-liposomes

After long PpIX-light interval (t_PpIX_ = 3 h), PpIX was illuminated inside the cells at the Q-band 630 nm at different radiant exposure doses of 134, 202, 403 and 672 mJ.cm^−2^. ROS generated by PDT process is causing irreversible damage to the tumor and the microvasculature that ensures a surplus of inflammatory and immune response leads to tumor regression (Wilson & Patterson, 2008; Johansson & Andersson-Engels, 2010; Rwei et al., 2015). Variable degrees of PpIX dose-response were observed, in response to the radiant exposure doses received and to the mole fraction of TELs incorporated in liposomes (Figure 2). In SKOV-3, TEL_9mol%_ liposomes revealed significant differences in viability till 25 µM PpIX as shown in Figure 2(a_1_). However, TEL_62mol%_ liposomes revealed significant differences at all concentrations applied (*p* < .01) (Figure 2(a_2_)). The yield of ROS production is directly proportional to the consumption of ambient oxygen upon illumination of PpIX and the radiant exposure dose applied. This can be seen from the results of *c*PDT after illumination. On the other hand, the results pointed out that prolonged release effect of TEL_62mol%_ provides better tuning of radiant exposure doses collectively with the applied PpIX doses. In SKOV-3, ROS enhancement ratio for PpIX in TELs-liposomes was determined to be 1.750 ± 0.087: 2.013 ± 0.018 at 134 mJ/cm^2^ for TEL_9mol%_ and TEL_62mol%_, respectively, compared to PpIX in DMSO. TEL_62mol%_ based liposomes yielded the highest ROS enhancement of 3.2 ± 0.152, 3.3 ± 0.155, 3.9 ± 0.246 and 4.321 ± 0.172 for 134, 202, 403 and 672 mJ.cm^−2^, respectively compared to non-irradiated cells (Figure 2(a_3_)). Still, a reduced cellular viability is most likely to be induced at lower radiant exposure doses without overwhelming the process of PDT by cellular oxygen depletion (Khaing Oo et al., 2012; Guo et al., 2018). Only under these circumstances, a sufficient yield of ROS can be deliberately modulated that can initiate mitochondrial dysfunction and promote cell death cascades. In Figure 2(B), the cellular photo responses to PpIX in L929 showed higher resistance to radiant exposure doses for TEL_9mol%_ liposomes (Figure 2(b_1_)) and TEL_62mol%_ liposomes (Figure 2(b_2_)) (*p* < 0.01) than that observed in SKOV-3 (Figure 2(A)). Thus, the impact of PpIX is more to be observed in SKOV-3 than in L929 cells. This may be attributed to different capacities in naturally existing antioxidant defense. The calculation of the PpIX_IC50_ values was also considered for direct comparison of PpIX photo responsiveness among TEL-liposomes and under comparable radiant exposure doses. The values of PpIX_IC50_ showed obvious inhibition of cellular viability at variable degrees.

### Cellular uptake of PpIX in TEL-liposomes

The ability of the photosensitizer to localize preferentially inside the cellular compartment makes it available to exert its cytotoxic effects (Soenen et al., 2011; Martens et al., 2014). Hence, PDT outcomes is strongly dependent on the uptake of the photosensitizer by tumor cells, a condition which is a prerequisite for an efficient PDT (Mehta et al., 1994; Li and Na, 2011; Zhen et al., 2013). Incubation of TEL_9mol%_ for 1 h showed obvious fluorescence intensity (Figure 3(A); magnified right panel, a_1_). The increase in cellular localization of PpIX is closely correlated to the enhanced *c*PDT in L929 cells as previously discussed. TEL_62mol%_ liposomes prevailed a lower degree in fluorescence intensity compared to TEL_9mole%_ liposomes owing to prolonged release effects of TEL_62mole%_ (Figure 3(B); magnified right panel, b_1_). Cells incubated with free PpIX showed intensified cellular toxicity as indicated by loss of their integral cellular morphology, cell nucleus and eventually cellular collapse, the signs that were observable after 1 h incubations (Figure 3(C); magnified right panel, c_1_). A time-dependent distribution pattern was not observed in case of TEL_9mole%_ liposomes after 3 h incubation (Figure 3(A); magnified right panel, a_2_). Indeed, TEL_62mole%_ liposomes showed distribution pattern after 3-h incubation indicating that PpIX has entered the cells via an endocytotic pathway and has co-localized preferentially inside the cellular compartment (Figure 3(B); magnified right panel, b_2_). The magnified right panel, b_2_ is further subjected to z-stack mode to show the co-localization and the distribution of PpIX throughout the cytoplasm. The de-quenching effect of PpIX throughout the cytoplasm became more intense as a function of time, suggesting that the intracellular effects led to PpIX release and the fluorescence intensity was restored to the ‘on’ state. Nevertheless, after 3-h incubation, free PpIX revealed a massive inherent toxicity. The cellular toxicity might be related to adsorptive mechanisms via a direct adhesion between free PpIX and extracellular membrane (Figure 3(C); magnified right panel, c_2_). Importantly, liposomes reduced the toxicity of PpIX itself. It has also been previously studied that the transport and release of lipophilic compounds from TELs based liposomes was carried out through lipid exchange which eventually led to fusion of TELs based liposomes with cellular membrane by which the lipophilic drug is able to diffuse from liposomal membrane to the cell membrane without the need of liposomal uptake into cells (Freisleben, 2000).

### *Vascular-targeted photodynamic therapy* (*vPDT) after short PpIX-light interval in CAM*

**Figure 4.**
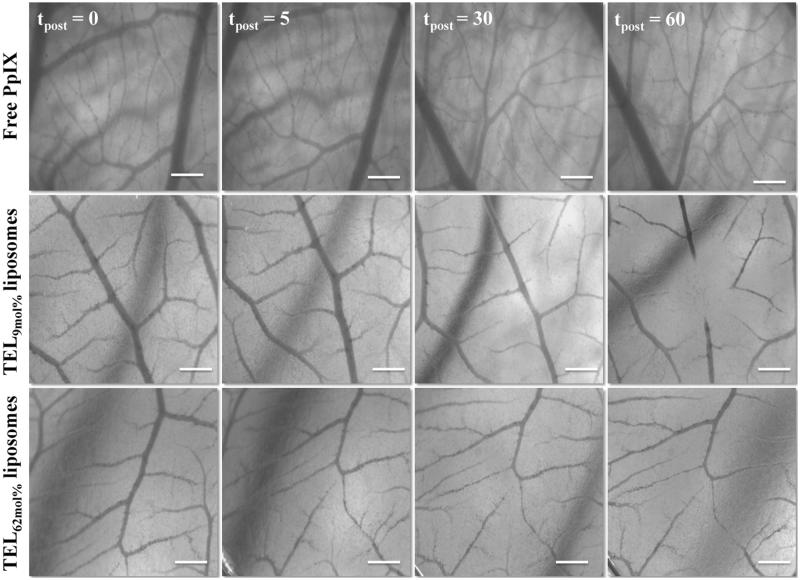
Stereomicrographs of CAM represent the occlusion of CAM vasculature mediated photodynamic therapy (*v*PDT). Image acquisitions were performed at 5 minutes before PDT (t_post_ 0) and at t_post_ 5, 30, and 60 minutes after PDT following IV-injection of free PpIX in 20% PEG 16000 containing HEPES-buffered saline, PpIX in TEL_9mol%_ or in TEL_62mol%_ liposomes at TL: PpIX ratios of 10. Bar represents 500 μm.

Changes in CAM microvasculature were monitored and scored after *v*PDT. Typical stereomicrographs of CAM vasculature injected with PpIX in TEL-liposomes are illustrated in Figure 4. CAM injected with sterile HEPES-buffered-saline or empty liposomes showed no obvious blood flow changes. The applied light dose did not provoke any changes in perfusion and/or integrity of CAM vasculature. Next, CAM injected with free PpIX showed level 0 response (t_post_ ≥ 60 minutes) after *v*PDT, representing intact and unaffected changes in- or outside the irradiated region. The effects on CAM following I.V. administration of PpIX in TEL-liposomes has confined within CAM vasculature. This was indicated by local destruction of small CAM capillaries (Ø ≤ 10 µm) within the irradiated area, at an onset of less than 60 minutes after laser irradiation. An effective closure of blood vessels was recorded after irradiation, which revealed dependency on TELs mole fraction in the liposomes. Vascular time-dependent profiles were most likely due to the retention of quenched PpIX in liposomes. Nevertheless, total CAM vascular (Ø ≤ 70–100 µm) closure and/or destruction was visible (Figure 4, t_post_ = 60) in case of TEL_62mol%_ as evident by disappearance of the arterioles and venules hierarchies; while most of the surrounding, healthy vasculature, remained functional until the completion of *v*PDT protocol. However, perfusion was ceased in the vasculature representing neither thrombosis nor hemorrhage. At a short PpIX-light interval, most PpIX was likely to be localized in CAM vasculature, while little PpIX had permeated and retained in the extravascular matrix and surrounding tissues. The results could be explained previously, where the massive accumulation of PpIX in the blood vessels and endothelial layers most particularly resulted in rearrangement of the cytoskeleton of endothelium, leading to damage of blood vessels accompanied eventually with thrombosis and micro vessel occlusion (Johansson & Andersson-Engels, 2010). A rapidly provoked thrombosis/hemorrhage and the collateral shutdown of the surrounding structures in- and outside the irradiated area were previously also reported in case of low mole fraction TELs based liposomes (Mahmoud et al., 2015, 2017). However, as vascular shutdown lead to hypoxia in tumor regions thereby the oxygen depletion might hamper the process of PDT completion. The optimal treatment resulted in damages ranging between 3 and 4 grading were observed as TELs concentration was increased. The extent of vascular damage induced by PpIX could be inferred that TELs seem to reduce the associated PDT photothrombic adverse event.

## Conclusions

In order to improve the monomerization of PpIX and hence its tumor accumulation, a subtle blend of formulation design based on tetraether lipids was developed. PpIX was confined in spherically stable TEL-based liposomes showing sustained release pattern and obvious stability in human serum. The prompt vascular structural changes *in-ovo* chick chorioallantoic membrane (CAM), which examined in real time, showed no collateral damage to quiescent vasculature. A reasonable mole fraction of TELs reduced the associated PDT photothrombic effect. Profoundly, TEls liposomes demonstrated potential PDT effect after long PpIX-light interval at different radiant energy doses at significant levels.

## Disclosure statement

No potential conflict of interest was reported by the authors.
